# Unleashing the Potential of Residual and Dual-Stream Transformers for the Remote Sensing Image Analysis

**DOI:** 10.3390/jimaging11050156

**Published:** 2025-05-15

**Authors:** Priya Mittal, Vishesh Tanwar, Bhisham Sharma, Dhirendra Prasad Yadav

**Affiliations:** 1 Chitkara University Institute of Engineering and Technology, Chitkara University, Rajpura 140401, Punjab, India; mittal.priya@chitkara.edu.in (P.M.); vishesh.tanwar@chitkara.edu.in (V.T.); 2 Centre of Research Impact and Outcome, Chitkara University, Rajpura 140401, Punjab, India; 3 Department of Computer Engineering & Applications, G.L.A. University, Mathura 281406, Uttar Pradesh, India

**Keywords:** ResNet50V2, ViT, hybrid model, dual-stream architecture, attention mechanism

## Abstract

The categorization of remote sensing satellite imagery is crucial for various applications, including environmental monitoring, urban planning, and disaster management. Convolutional Neural Networks (CNNs) and Vision Transformers (ViTs) have exhibited exceptional performance among deep learning techniques, excelling in feature extraction and representational learning. This paper presents a hybrid dual-stream ResV2ViT model that combines the advantages of ResNet50 V2 and Vision Transformer (ViT) architectures. The dual-stream approach allows the model to extract both local spatial features and global contextual information by processing data through two complementary pathways. The ResNet50V2 component is utilized for hierarchical feature extraction and captures short-range dependencies, whereas the ViT module efficiently models long-range dependencies and global contextual information. After position embedding in the hybrid model, the tokens are bifurcated into two parts: q1 and q2. q1 is passed into the convolutional block to refine local spatial details, and q2 is given to the Transformer to provide global attention to the spatial feature. Combining these two architectures allows the model to acquire low-level and high-level feature representations, improving classification performance. We assess the proposed ResV2ViT model using the RSI-CB256 dataset and another dataset with 21 classes. The proposed model attains an average accuracy of 99.91%, with precision and F1 score of 99.90% for the first dataset and 98.75% accuracy for the second dataset, illustrating its efficacy in satellite image classification. The findings demonstrate that the dual-stream hybrid ResV2ViT model surpasses traditional CNN and Transformer-based models, establishing it as a formidable framework for remote sensing applications.

## 1. Introduction

Remote sensing plays a crucial role in monitoring Earth’s dynamic environment, enabling precise observations of natural and anthropogenic changes over time. Advances in satellite and aerial imaging technologies have facilitated the acquisition of high-resolution spatial, spectral, and temporal data, with widespread applications in urban development, deforestation monitoring, disaster assessment, and agricultural management [1]. A fundamental task in remote sensing is change detection (CD), which involves identifying differences between time-stamped images of the same geographical area [2]. Accurate CD is essential for understanding environmental transformations and supporting informed decision-making, yet it remains challenging due to the complexities of remote sensing imagery, such as high dimensionality, varying illumination, sensor noise, and heterogeneous land cover.

Traditional CD methods, including pixel-based, object-based, and shallow machine learning approaches [3], have shown limited effectiveness. Pixel-based techniques are sensitive to noise and lack contextual understanding, while object-based methods, though more accurate, are computationally intensive and parameter-dependent. Shallow machine learning approaches improve performance but struggle to capture hierarchical features and long-range dependencies [4].

Recent deep learning models, particularly Convolutional Neural Networks (CNNs) like ResNet, have significantly advanced CD by automatically learning spatial feature hierarchies. However, CNNs are limited by their local receptive fields, which restrict their ability to model global context, crucial for large-scale remote sensing data. Vision Transformers (ViTs), with their self-attention mechanisms, offer a powerful alternative by capturing long-range dependencies and global contextual information. Nevertheless, ViTs require large-scale data and substantial computational resources to perform effectively.

To address these challenges, this paper proposes a novel dual-stream hybrid model, which has the following contributions:

(1) We utilized ResNet50V2 for high dimensional spatial features that enhanced the remote image analysis performance.
(2) The feature map obtained from the residual block is passed to a ViT module, where patch-wise embeddings allow the model to learn global contextual relationships through self-attention mechanisms.
(3) To achieve local and global attention, we divided the query (Q) into two parts (q1 and q2). We passed to the dual-stream, which enhanced the model’s focus on the edge and boundary region of the complex, multi-temporal, and multispectral satellite imagery.

By combining the strengths of CNNs and ViTs, the proposed ResV2ViT model achieves improved accuracy, robustness, and scalability, while mitigating issues such as sensor noise, varying illumination, and high data dimensionality.

The remainder of the paper is structured as follows: Section 2 reviews traditional, CNN-based, and Transformer-based change detection methods. Section 3 presents the proposed ResV2ViT architecture. Section 4 details the experimental setup, including datasets, metrics, and implementation. Section 5 discusses the results and compares the proposed model with state-of-the-art techniques. Finally, Section 6 concludes the paper and outlines directions for future research.

## 2. Related Work

Sun et al. [5] implemented a fast MODA (motion object-detection algorithm) on constrained hardware. He applied the sentinel-2 Imagery Model dataset. This model attained an OA of 93%, an F1 score of 90%, and an IoU of 82%, facilitating accurate environmental monitoring. Wang et al. [6] specialized in radar-based object detection; RODNet (Radar Object-Detection Network) demonstrated a precision of 86% and recall of 88%, contributing to advancements in autonomous sensing technologies. Zhang et al. [7] designed SATNet (Spectrum-Aware Transform Network) for spectral awareness in remote sensing; SATNet achieved an overall accuracy (OA) of 98.39%, Kappa coefficient of 0.8883, precision of 83.02%, and F1 score of 89.69%. Shi et al. [8] utilized DAHT-Net (Deformable Attention-Guided Hierarchical Transformer Network) deformable attention mechanisms; DAHT-Net achieved a precision of 92.49%, recall of 93.18%, F1 score of 92.83%, intersection over union (IoU) of 92.4%, and OA of 98.93%, indicating robust performance in complex detection tasks. Gao et al. [9] focused on spatial feature transformation. DSFT-Net (Deep Spatial Feature Transformation Network) reported a mAP of 91.63%, enhancing detection accuracy in spatially complex scenarios.

Butler et al. [10] extended Faster R-CNN with instance segmentation; Mask R-CNN achieved an overall detection accuracy exceeding 80%, enhancing object detection and segmentation tasks. Xu et al. [11] introduced a new model, TCIANet (Transformer-Based Context Information Aggregation Network), which uses a filter-based visual tokenizer and feature fusion module. The model showed a precision of 95.98%, recall of 93.41%, F1 score of 95.68%, IoU of 92.57% and OA of 98.73%. Jiang et al. [12] proposed a lightweight, full-scale hybrid network named LFHNet. The network comprises CNN, multilayer perceptron (MLP), and Transformer. The model showed IoU as 82.27% and OA as 99.03%. Han et al. [13] designed a discriminative Siamese network, hierarchical attention network (HANet), which can integrate multiscale features and refine detailed features. The model showed the OA as 99.16% and IoU as 78.82%. Wan et al. [14] introduced the Category Context Learning-based Difference Refinement Network (CLDRNet), which addresses semantic content discrepancies from the CD perspective. The model achieved and IoU of 92.12% and OA of 99.03%.

Lie et al. [15] introduced MDEFNet (a multiscale difference feature enhancement network) to extract the most discriminative features from bitemporal remote sensing images. The model showed an accuracy of 91.06% and an IoU of 68.29%. Sun et al. [16] introduced STCD Former to interact with the patch token to learn the change rules. The model showed overall accuracy of 99.25% and Kappa as 95.99%. Jin et al. [17] integrated CR-DINO (Camera Radar-based DETR) camera and radar data; CR-DINO reported a mean average precision (mAP) of 38.0 and 41.7 across different evaluation settings, enhancing object detection capabilities.

Jia et al. [18] combined Transformer and CNN architectures. This hybrid model reached a mean IoU (MioU) of 82.41% after extensive training, indicating effective feature integration. Guo et al. [19] proposed TFIFNet (Transformer with feature interaction and fusion network) for CD (change detection). The model showed a precision of 90.87%, recall of 87.08%, F1 score of 88.77%, IoU of 80.39%, and OA of 92.29%.

Tan et al. [20] proposed BD-MSA, the model for object detection that effectively delineates the change area’s boundary information while separating the change region’s main body from its boundary. The model recorded a precision of 88.01% and an F1 score of 83.98%. Xiong et al. [21] suggested MLA-Net, an improved model that is presented to delineate feature distinctions and mitigate the influence of extraneous alterations due to intricate backgrounds. The model recorded an accuracy of 99.08% and an IoU of 83.27%. Table 1 summarizes the recent works for satellite image classification.

## 3. Methodology

This section highlights the framework for the ResV2ViT model’s execution, including the specification of data gathering, model architecture, and training methodologies. It emphasizes the integration of sophisticated deep learning methodologies, including Transformer-based and convolutional networks, to enhance the accuracy of feature extraction and classification in remote sensing image analysis.

### 3.1. Dataset

Developing robust AI-driven models for identifying remote sensing images relies on high-quality benchmark datasets that offer diversity, scalability, and accurate annotations. Inspired by large-scale datasets like Million-AID, RSI-CB256 is vital in deep learning-based RS applications, enhancing land cover classification, environmental monitoring, and geospatial analysis.

**Figure 1:**
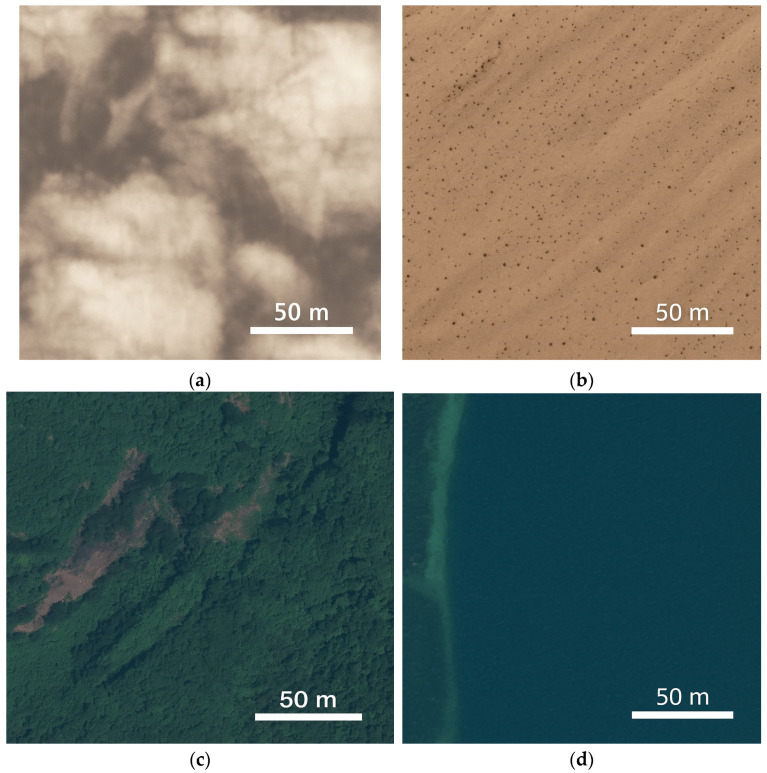
RSI-CB256 dataset sample images: (**a**) cloudy, (**b**) desert, (**c**) forest, and (**d**) water.

Two datasets are used while developing the classification model. The first dataset, the Satellite Image Classification Dataset-RSI-CB256, is a benchmark dataset designed for RS image classification, comprising 5631 images across four categories: green_area, desert, cloudy, and water. The images are sourced from satellite sensors and Google Maps snapshots. Each class contains 1500 images, except the dessert category, which has 1131 images. Collectively, the dataset has a total size of 22.58 MB. Designed for 5-fold cross-validation, RSI-CB256 supports robust model evaluation, making it a valuable resource for RS image analysis and AI research. The sample images of each class, cloudy, desert, forest, and water, are shown in Figure 1.

**Figure 2:**
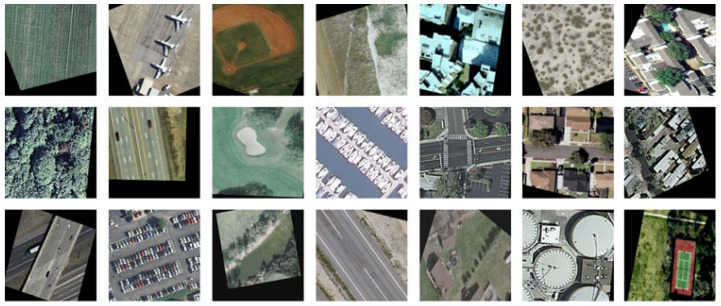
Land Use Scene Classification Dataset sample images.

The second dataset, the Land Use Scene Classification Dataset, was obtained from UC Merced. It contains satellite images of 21 classes, such as agricultural, airplane, baseballdiamond, beach, buildings, chaparral, denseresidential, forest, freeway, golfcourse, harbor, intersection, mediumresidential, mobilehomepark, overpass, parkinglot, river, runway, sparseresidential, storagetanks, and tenniscourt. The original size of the images is 256 × 256 pixels. Initially, there were 100 images per class with a spatial resolution of 1 foot/pixel. After augmenting each image 4 times, including random rotations (±30°), horizontal and vertical flipping, random cropping, brightness adjustment, and slight color jittering, the size of each class was brought up to 500 images. This allowed for making a more robust model. Collectively, the dataset was 2 GB in size. The sample images of the dataset are shown in Figure 2.

### 3.2. Proposed Method

**Figure 3:**
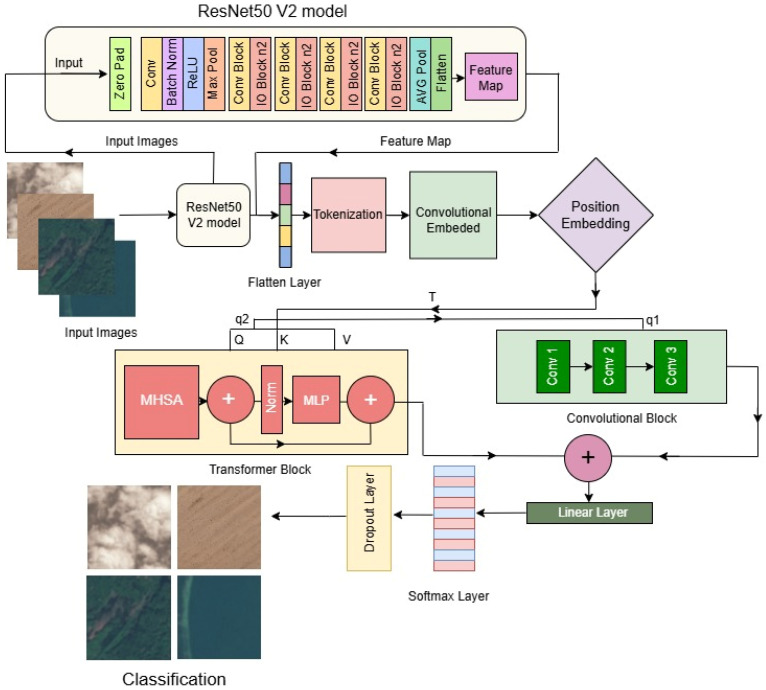
Model diagram of proposed ResV2ViT model.

Figure 3 illustrates the architecture of the ResV2ViT model, integrating ResNet50 V2 and Vision Transformer (ViT) for remote sensing image classification. The ResNet50 V2 module extracts hierarchical local features from input images, producing a feature map. These features are then tokenized and embedded before undergoing position encoding. The ViT module, equipped with multi-head self-attention, captures global contextual relationships. The combined features pass through convolutional layers and a linear classifier for final prediction, leveraging both local and global feature representations.

#### 3.2.1. Feature Extraction Using ResNet50 V2

The ResNet-50 component in the hybrid model functions as an effective feature extractor, acquiring low-level spatial details like textures and edges, as well as high-level patterns essential for differentiating various land cover types in remote sensing satellite imagery. This hierarchical feature extraction produces a detailed feature map that incorporates low-, mid-, and high-level abstractions, guaranteeing a thorough representation of the satellite image. The residual unit is:

(1) $$y_{1}=h(x_{1})+F(x_{l},\;W_{l})$$

(2) $$x_{l+1}=f(y_{l})$$

where $h(x_{l})=x_{l}$ is an identity mapping. ResNeXt employs grouped convolutions, aggregating multiple transformation paths:

(3) $$F_{l}=F_{l-1}+\sum_{i=1}^{C}T_{i}(F_{\left(l-1\right)})\;$$

#### 3.2.2. Tokenization of the Feature Map

ResNet-50 v2’s multi-scale features are extracted and then combined into a dense feature map that preserves the fine details required for exact classification. The dual-stream Vision Transformer (ViT) receives this fused feature map tokenized as input. Using a learnable linear projection, the tokenization method flattens the feature map into non-overlapping patches and maps them into an embedding space.

(4) $$T_{i}=W_{e}*Flatten(F_{i})+b_{e}\;$$

where $T_{i}$ is the $i^{th}$ token. $W_{e}$ is the learnable projection matrix. $b_{e}$ is the bias term. $F_{i}$ is the corresponding patch extracted from the feature map F.

After tokenization, each patch is transformed into a one-dimensional vector representation:

(5) $$T_{i}^{1D}=Flatten(T_{i})+P_{e}$$

where $T_{i}^{1D}$ is the one-dimensional token representation and $P_{e}$ is the position embedding added to the token.

#### 3.2.3. Split Query into Two Parts

In the classical Transformer, after tokenization, three vectors, Q (query), Key (K), and V (value), are calculated. After that, attention is calculated by the MHSA (multi-head self-attention) using the whole Q. Due to this, it takes quadratic time for the attention mechanism. In addition, it only provides global contextual information. In the proposed study, features obtained from the ResNet50V2 are flattened into a 1D feature map. After that, $Q\in R^{P\times C}$. Here, P is the total pixels and C is the number of channels, which are partitioned into $q_{1}\in R^{P\times C_{1}}$ and $q_{2}\in R^{P\times C_{2}}$ along the channel dimension as follows.

$$q_{1}=T[:,0:C_{1}],q_{2}=T[:,C_{1},C_{2}]$$

where $C_{1}+C_{2}=C$; then, q1 is fed into the to the convolution block and q2 into the ViT encoder for the local and global spatial contextual information.

#### 3.2.4. Convolutional Block

The convolutional block processes q1 through additional convolutional layers. The convolutional stream generates complex textures, preserves vital boundaries including roadways, water bodies, and plant patches, and enhances local spatial information. This stream’s convolutional handling follows:

(6) $$T_{q1}^{\prime }=\sigma (W_{c}+T_{q1}+b_{c})$$

where $T_{q1}^{\prime }$ is the refined token representation. $W_{c}\;and\;b_{c}$ are the convolutional layer parameters, and σ is the activation function.

#### 3.2.5. Transformer Block

Simultaneously, the Transformer block processes q2 by applying multi-head self-attention (MHSA) to model global spatial dependencies, learning intricate relationships between different land cover regions within the image. Further, q2 is transformed into query, key, and value matrices: $Q=W_{q}.q_{2},\;K=W_{k}.q_{2},\;V=W_{v}.q_{2}$, where Wq, Wk, and Wv are the learnable weight matrices. The self-attention mechanism in the Transformer stream is computed as:

(7) $$Attention(T_{q_{2}})=softmax(\frac{QK^{T}}{\sqrt{d_{k}}})V\;$$

where dk is the scaling factor to normalize the dot-product attention scores. The softmax function ensures proper weight distribution across attention scores.

This allows the model to identify spatial dependencies among distant parts of the image, which is essential for accurately classifying large-scale remote sensing imagery.

#### 3.2.6. Final Feature Fusion and Classification

The most efficient feature representation is achieved by integrating the convolutional and Transformer streams and uses Global Average Pooling (GAP):

(8) $$Z=GAP(T_{q_{1}}^{\prime }+Attention(T_{q_{2}}))\;$$

The classification head applies a fully connected (FC) layer followed by a softmax activation:

(9) $$P=Softmax(\;W_{fc}Z+b_{fc})\;$$

where Z is the final feature representation. $W_{fc}\;and\;b_{fc}$ are the classification layer parameters and P represents the predicted class probabilities.

#### 3.2.7. Loss Function

We utilize the categorical cross-entropy loss function to optimize the hybrid ResV2ViT model, a standard approach for multi-class classification tasks. This loss function quantifies the divergence between the projected probability distribution and the actual label distribution, ensuring that the model learns to allocate high probabilities to the proper classes. The categorical cross-entropy loss is articulated as follows:

(10) $$L=\sum_{i=1}^{C}y_{i}\;log(y_{p}i)$$

where *C* is the total number of classes, $y_{i}$ is the true class label, and $y_{p}$ is the predicted probability for class i. $y_{p}$ can be computed as:

(11) $$y_{p}=\frac{e^{z_{i}}}{\Sigma_{j=1}^{C}e^{z_{j}}}$$

where $z_{i}$ represents the output logit from the final classification layer before applying the softmax function.

This hybrid ResV2ViT model enables the ability to analyze satellite images at both the micro and macro levels, allowing for the precise distinction between different land cover types, urban structures, and natural formations. Integrating local feature extraction from CNNs and global feature modeling from Transformers enhances the classification of satellite imagery, making the model highly effective for remote sensing applications. Figure 3 shows the model diagram of the proposed ResV2Vit model. The algorithm of the proposed method is described in the Section 3.3.

### 3.3. Algorithm 1: ResV2ViT for Satellite Image Analysis

We summarized the abbreviations used in the proposed study in Table 2.
**Algorithm 1.** Algorithm of the proposed method for satellite image analysis**Input:** Remote sensing Satellite Image X**Output:** Predicted land cover class P.        (1)Initialize ResNet-50 model. Process input image X through convolutional layers:$\begin{array}{c} \;\;\;\;\;\;\;\;\;\;\;\;\;\;\;\;\;\;\;\;\;\;\;\;F=ResNet(X)\; \end{array}$ where *F* is the extracted feature map containing low-, mid-, and high-level spatial features.        (2)Divide *F* into non-overlapping patches *F_i_*. Flatten and linearly project each patch into token representation:$\begin{array}{c} \;\;\;\;\;\;\;\;\;\;\;\;\;\;\;\;\;\;\;\;\;\;\;\;T_{i}=W_{e}*Flatten\;(F_{i})+b_{\;e\;} \end{array}$ Add position embeddings to preserve spatial relationships: $\begin{array}{c} \;\;\;\;\;\;\;\;\;\;\;\;\;\;\;\;\;\;\;\;\;\;\;\;T_{i}^{1D}=Flatten(T_{i})+P_{e}\; \end{array}$        (3)Split tokenized features into two separate streams:$\begin{array}{c} \;\;\;\;\;\;\;\;\;\;\;\;\;\;\;\;\;\;\;\;\;\;\;\;T_{conv}=T_{\left[0:\frac{n}{2}\right]}^{1D}\; \end{array}$ $\begin{array}{c} \;\;\;\;\;\;\;\;\;\;\;\;\;\;\;\;\;\;\;\;\;\;\;\;T_{trans}=T_{\left[\left(\frac{n}{2}\right):n\right]}^{1D}\; \end{array}$        (4)Process $T_{conv}$ through additional convolutional layers to refine spatial features:$\begin{array}{c} \;\;\;\;\;\;\;\;\;\;\;\;\;\;\;\;\;\;\;\;\;\;\;\;T_{i}^{\prime }=\sigma (W_{c}+T_{conv}+b_{c})\; \end{array}$        (5)Compute self-attention for $T_{trans}$:$\begin{array}{c} \;\;\;\;\;\;\;\;\;\;\;\;\;\;\;\;\;\;\;\;\;\;\;\;Attention(T_{trans})=softmax(\frac{QK^{T}}{\sqrt{d_{k}}})V\; \end{array}$ where *Q*, *K*, *V* are query, key, and value matrices.        (6)Fuse features from both streams using Global Average Pooling (GAP):$\begin{array}{c} \;\;\;\;\;\;\;\;\;\;\;\;\;\;\;\;\;\;\;\;\;\;\;\;Z=GAP(T^{\prime }+Attention(T))\; \end{array}$ Apply a fully connected (FC) classification layer with softmax activation: $\begin{array}{c} \;\;\;\;\;\;\;\;\;\;\;\;\;\;\;\;\;\;\;\;\;\;\;\;P=Softmax\;(\;W_{fc}Z+b_{fc})\; \end{array}$

## 4. Results

In this section, we have discussed the experimental results of the ResV2ViT proposed model on both datasets. Firstly, experimental settings are discussed, followed by confusion matrices for each of the five folds for Dataset 1 and confusion matrix for 21 classes of the second dataset. Then, the comparison table of confusion matrices for both datasets is also discussed.

### 4.1. Experimental Settings

The script for the proposed approach is developed in Python 3.9, utilizes TensorFlow 2.0, and operates on a Windows 11 PC manufactured iby Dell company situated in Texas, United State equipped with an Nvidia GeForce GTX TITAN X GPU and 128 GB of RAM manufactured by Nvidia corporation Calfornia, United State. The batch size, epoch, and learning rate are set to 8, 50, and 0.0001, respectively. The dropout function was set to 0.3. The Adam Optimizer reduces the loss function and improves model training. Given the dataset’s asymmetry, we utilized fivefold cross-validation in Dataset 1 to prevent biased performance assessments. In each cycle of cross-validation, 80% of the images are used for training, and the remaining 20% are designated for validation.

### 4.2. Quantitative Results

We assessed the proposed model by 5-fold cross-validation on the RSI-CB256 dataset and confusion matrix of 21 classes of the second dataset. The images in the datasets are extracted from large images obtained from the USGS National Map Urban Area and stored in the RGB format. The RGB images have three channels; therefore, we avoid the dimensional reduction techniques. Moreover, the Indian Pines, Pavia, or Salinas datasets are obtained by the sensors and stored in the. mat file format having more than 100 bands.

#### 4.2.1. For RSI-CB256 Dataset

**Figure 4:**
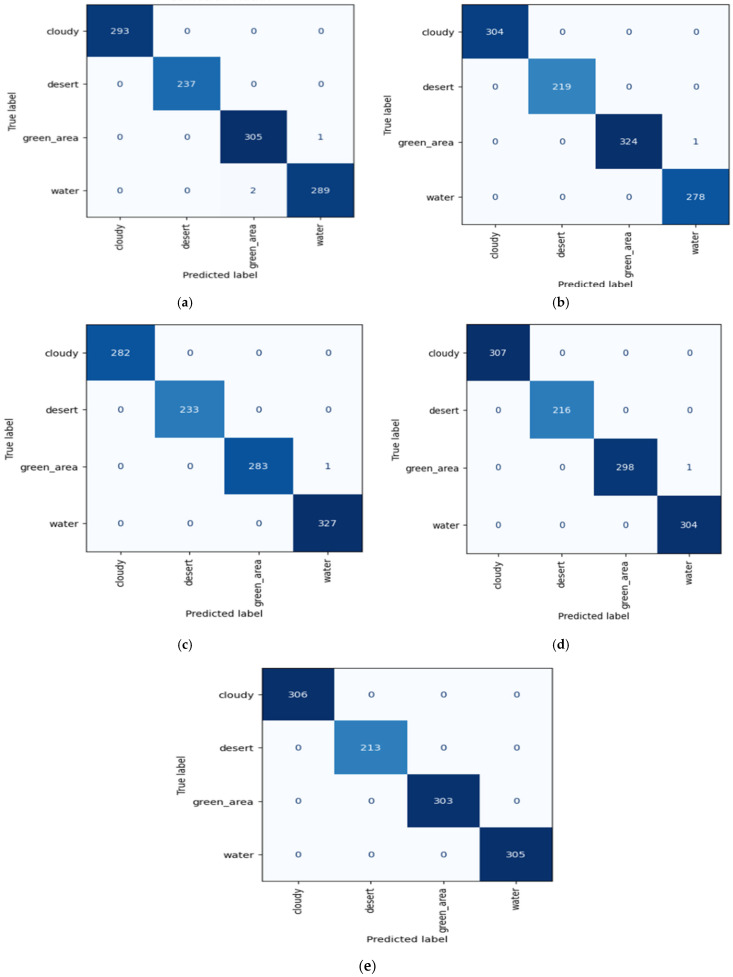
Confusion matrices obtained from each of the 5-fold cross-validations.

The outcomes of each fold for Dataset 1 are illustrated in Figure 4. The performance varies among folds, indicating the model’s strengths and weaknesses. Table 3 displays the performance metrics of the ResV2ViT model using the RSI-CB256 dataset for classifying remote sensing photos into four categories: cloudy, desert, forest, and water.

For fold 5, ResV2ViT produced zero false positive and zero false negative values depicting high accuracy. ResV2ViT produced one false positive for fold 1, fold 2, fold 3, and fold 4. Also, the proposed model produced two false negative values in fold one. The overall classification performance of the ResV2ViT model on the RSI-CB256 dataset shows an average precision of 99.90%, recall of 99.90%, and F1 score of 99.90%, confirming its high reliability. The average accuracy of 99.91% suggests excellent model generalization, with folds 5, 4, and 3 achieving over 99% accuracy. Overall, the ResV2ViT model demonstrates robust performance, with minimal misclassification rates and high consistency across all folds.

#### 4.2.2. For Land Use Scene Classification Dataset

**Figure 5:**
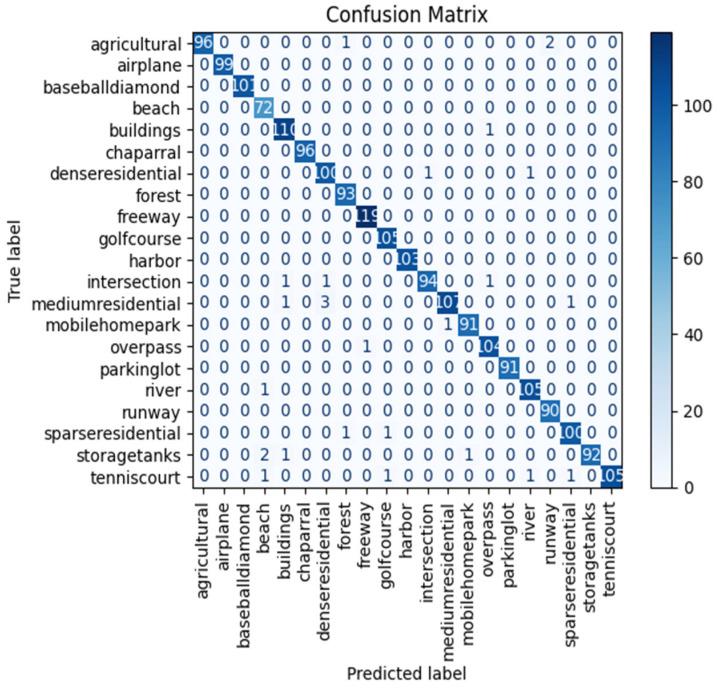
Confusion matrix obtained for second dataset.

Furthermore, we applied the proposed ResV2ViT model on the second dataset, which contains 21 classes such as agricultural, airplane, beach, etc. The ResV2ViT model is trained for 50 epochs, and the confusion matrix is obtained by accessing the 21 classes, as shown in Figure 5. High diagonal values like 103, 105, 119, 96, and 99 represent correct predictions of most cases for each subtype. Off-diagonal values signify misclassifications that are very small in number. Like, in the agriculture row, one case is misclassified as forest and two are misclassified as runway. Similarly, 1 case is misclassified as buildings, 3 are misclassified as denseresidential, and 1 case is misclassified as sparseresidential in mediumresidential row, whereas 107 cases are correctly classified as mediumresidential. Thus, the proposed ResV2ViT model classified the subtypes with remarkable accuracy and minimum errors.

Table 4 displays the performance metrics of the ResV2ViT model using the Land Use Scene Class dataset to classify the images into 21 subtypes using parameters like precision, recall, F1 score, and accuracy. The model showed high values of performance parameters, such as 98.69% precision, 98.75% recall, 98.71% accuracy, and an F1 score, indicating highly accurate classification.

## 5. Discussion

This section evaluates the efficacy of our proposed hybrid ResV2ViT model on the RSI-CB256 satellite image classification dataset. The evaluation initiates, with confusion, matrices for each fold, offering comprehensive information about class-specific performance. Furthermore, we evaluate model efficacy through training and validation accuracy curves and training and validation loss curves to monitor learning progression and convergence. The ROC curve is also generated to evaluate the model’s classification efficacy across several classes. The performance indicators collectively underscore the resilience and precision of the ResV2ViT model in satellite image classification.

### 5.1. Accuracy and Loss Curve

**Figure 6:**
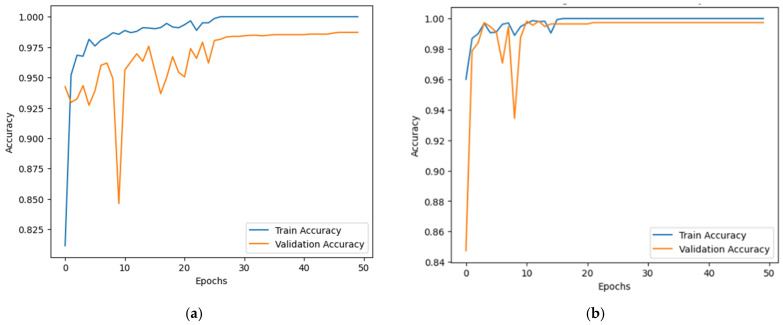
Accuracy over training and validation on (**a**) Dataset 1 and (**b**) Dataset 2.

Emphasizing loss reduction and accuracy improvement, Figure 6 shows the proposed model’s training and validation accuracy curves throughout 50 epochs for both datasets. The first graph shows the accuracy curve for the RSI-CB256 dataset, and the second graph shows the accuracy curve for the second dataset having 21 classes. The y-axis shows accuracy; the x-axis relates to the epoch count. The blue line demonstrates training accuracy; the orange line shows validation accuracy. Training accuracy rapidly increases, reaching nearly 100%, indicating effective learning. Validation accuracy also improves but fluctuates in the early epochs, showing generalization challenges. In the first dataset, accuracy stabilizes quickly, while in the second, minor overfitting occurs with slight validation dips. Around epoch 10, fluctuations reduce, and validation accuracy stabilizes between 97–99%. By epoch 20, both curves show minimal variation, indicating intense learning. Despite early instability, the model generalizes well, achieving high accuracy. Regularization could further improve generalization and prevent overfitting.

**Figure 7:**
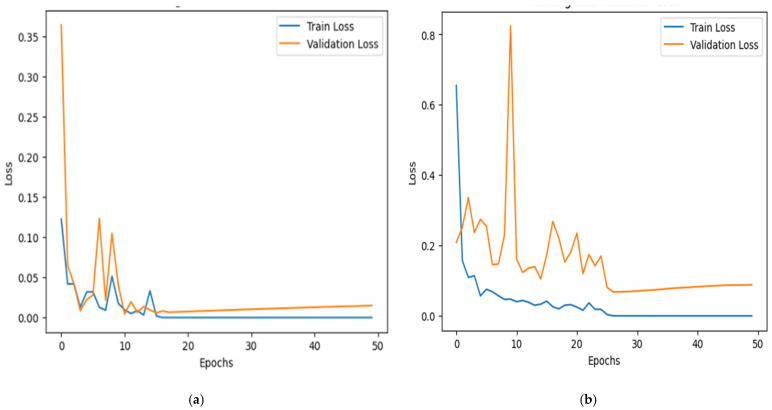
Loss over training and validation on (**a**) Dataset 1 and (**b**) Dataset 2.

Figure 7 demonstrates the training and validation loss curve for the two datasets. The y-axis represents cross-entropy loss, while the x-axis denotes the number of epochs. The blue line represents training loss, and the orange line represents validation loss. Initially, both losses are high but decrease rapidly within the first 10 epochs. The validation loss fluctuates before stabilizing after 20 epochs, while training loss continues to decline steadily. In the second graph, validation loss exhibits sharp spikes, suggesting possible overfitting. The increasing trend in validation loss after 30 epochs indicates that the model may not generalize well. Regularization techniques such as dropout and early stopping could improve stability and reduce overfitting. Specifically, early stopping was implemented with a patience of 5 epochs, and training was halted at epoch 45 (out of a maximum of 50), as no improvement was observed in the validation loss. Additionally, we applied dropout (rate = 0.3) in the classification head and L2 weight decay (λ = 1 × 10^−4^) across convolutional and Transformer layers. These regularization strategies collectively helped to improve the model’s generalization and prevent overfitting, as supported by the smoother validation loss curves in Figure 7.

The model generally shows excellent learning ability, with loss reduction and accuracy enhancement noted during training. Still, the variations in validation loss and a small gap between training and validation accuracy point to possible overfitting. Using these techniques, the model can avoid memorizing the training data and enhance its performance on test data.

### 5.2. Receiver Operating Characteristic Curve (ROC)

The images illustrate the Receiver Operating Characteristic (ROC) curve, assessing the classification efficacy of a model across many categories, i.e., four subcategories for Dataset 1 and 21 classes for Dataset 2. The ROC curve illustrates the True Positive Rate (TPR) about the False Positive Rate (FPR), showcasing the model’s capacity to differentiate between various classes.

**Figure 8:**
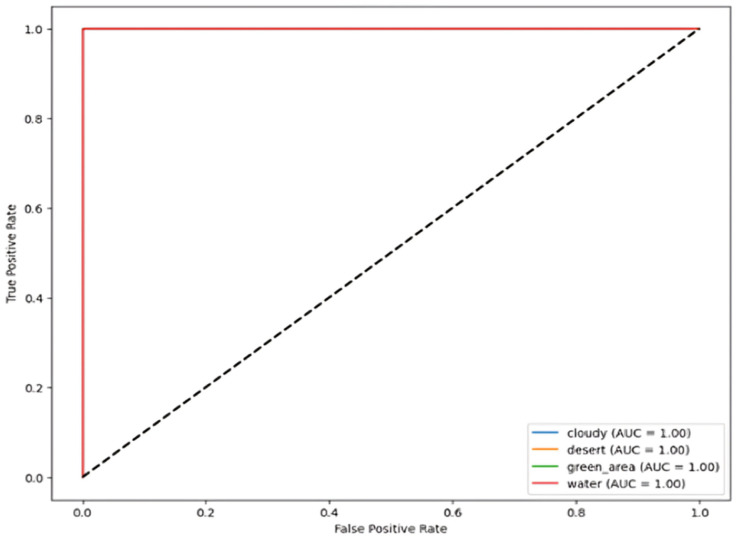
ROC curve for RS image classification dataset over four categories.

**Figure 9:**
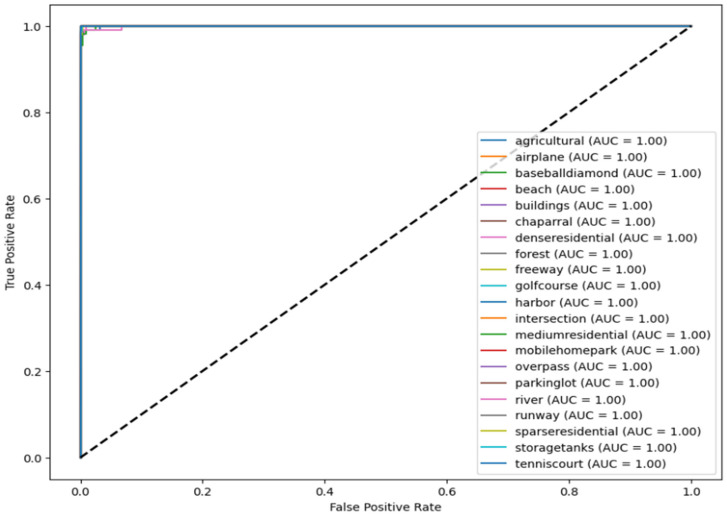
Roc curve for second dataset over 21 classes.

In Figure 8 and Figure 9, each colored line represents a distinct class, and the Area Under the Curve (AUC) for all classes equals 1.00, signifying flawless classification. AUC values go from 0 to 1, where 1.00 signifies a flawless model that accurately distinguishes between positive and negative cases without error. The black dashed diagonal line represents a random classifier with an AUC of 0.5, indicative of the worst-case situation.

Since all ROC curves attain the upper left corner (TPR = 1, FPR = 0), the model demonstrates flawless classification ability, devoid of false positives or false negatives. This indicates a model that is both highly accurate and well trained.

### 5.3. Comparison with STATE–of-the-Art (SOTA) Methods

The comparison of various deep learning models on the RSI-CB256 dataset reveals significant advancements in classification accuracy and performance metrics. The comparison of different models is shown in Table 5. ResNet50, widely used across multiple studies, demonstrated high accuracy, with Jayasree et al. [22] achieving 96.53%, Li et al. [23] recording 95.02%, and Scott et al. [24] attaining an impressive 99.38%. VGG-based models also performed well, with Yogesh et al.’s [25] VGG-16 achieving 99% accuracy and Kaur et al.’s [26] VGG-19 yielding 93% accuracy and a 93.25% F1 score.

Hybrid and advanced models further improved performance metrics. Tumpa et al.’s [27] LPCNN-SVM achieved the highest accuracy of 99.8%, alongside a precision of 99.67%. Similarly, in Ulla et al. [28], SATNet reached 99.15% accuracy with an F1 score of 99%, highlighting its robustness. Tehsin et al. [29] utilized a ResNet variant, achieving 97.7% accuracy and a 96.9% Kappa score. Sharma et al. [30] combined EfficientNet with SVM, achieving a 97.11% accuracy.

**Figure 10:**
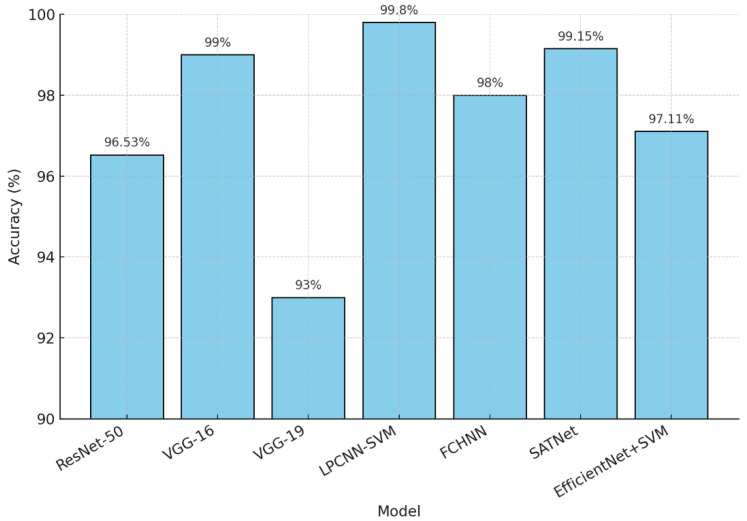
Comparison of various models with the RSI-CB256 dataset.

Liu et al. [31] utilized FCHNNN and achieved 98% accuracy. Overall, the RSI-CB256 dataset has proven effective for benchmarking various architectures, with hybrid models and advanced CNN variations consistently outperforming traditional architectures. A comparison of multiple models with the RSI-CB256 dataset is shown in Figure 10.

**Figure 11:**
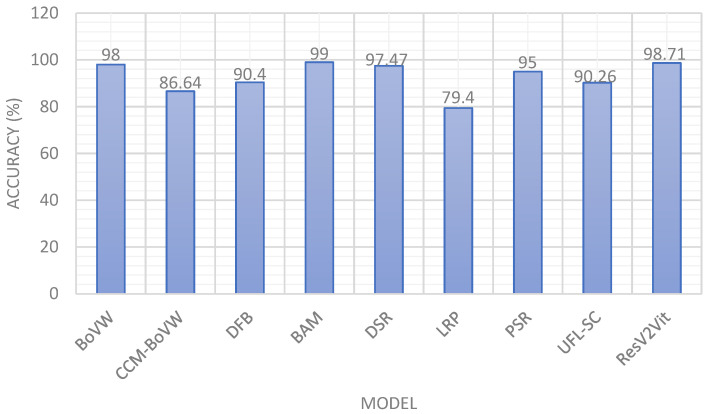
Comparison of various models with the Land Use Scene Classification dataset.

Also, a comparison of various models on the Land Use Scene Classification Dataset containing 21 classes is discussed in Table 6. Qi et al. [32] used the BoVW model attaining an accuracy of 98%. Zhao et al. [33] utilized a hybrid CCM-BoVW model recording an accuracy of 86.64%. Wu et al. [34] also used the Land Use Scene Classification Dataset on the Deep Filter Banks model with an accuracy of 90.40%. Li et al. [35] proposed the Best Activation Model and recorded an accuracy of 99%. Hu et al. [36] employed DSR with an accuracy of 97.47%. Yao et al. [37] recorded 79.40% accuracy with Large Random Patch (LRP). Fan et al. [38] also contributed using Unsupervised Feature Learning with an 89.05% accuracy. Chen et al. [39] used the PSR model, yielding an average accuracy of 95%. Finally, Hu et al. [40] employed the UFL-SC model, recording an accuracy of 90.26%. The graphical comparison of various models with the Land Use Scene Classification Dataset is depicted in Figure 11.

### 5.4. Comparison with Other Methods

Song et al. [41] designed a wavelet attention ResNeXt model to improve Convolutional Neural Networks’ extraction and generalization ability for detail and texture features. The developed model recorded an accuracy of 94.12%. Abba et al. [42] tested the Inception V4 method to suitably classify remote sensing satellite images into oil spill and no spill. The model recorded accuracy, precision, recall, and F1 scores, each of which was 96.98%. Saetchnikov et al. [43] demonstrated the YOLOV9 model on the Airbus dataset for aircraft detection and showed an average precision of 98.7%. Le et al. [44] identified Vit as the most suitable model for land-use classification for onboard-satellite remote sensing images. The model was chosen as the best, recording an accuracy of 98.76%. Huang et al. [45] proposed a novel SwinT method to capture 3D properties of hyperspectral images. The model recorded an accuracy of 80.15% on the DFC2018 dataset. Table 7 shows the comparison of various models on different datasets.

### 5.5. Ablation Study

To analyze the effectiveness of the proposed ResV2ViT hybrid model, an ablation study was conducted by evaluating different configurations: standalone ResNet50 V2, standalone Vision Transformer (ViT), their combination without dual processing, and the final proposed model. The results, summarized in Table 8, demonstrate the significant impact of integrating both architectures. The ResNet model leverages convolutional layers to extract local spatial features, achieving an accuracy of 98.75%, an F1 score of 98.57%, and a Kappa coefficient of 97.90%. However, its limited global context understanding restricts its classification performance.

(12) $$M_{ResNet}=f_{CNN}(X)$$

The ViT model captures long-range dependencies using self-attention mechanisms, resulting in improved performance with 99.10% accuracy, 99.05% F1 score, and a Kappa coefficient of 98.50%. However, ViT alone struggles with fine-grained spatial feature extraction.

(13) $$M_{ViT}=F_{transformer}(X)$$

The ResV2ViT hybrid model without dual processing combines both feature extraction techniques, leading to 99.45% accuracy, 99.40% F1 score, and a Kappa coefficient of 99.10%, demonstrating substantial improvement.

(14) $$M_{hybrid}=f_{CNN}(X)+f_{transformer}(X)$$

The proposed ResV2ViT hybrid model with dual processing further enhances spatial and contextual feature learning by integrating a specialized dual-processing mechanism. It achieves a 99.91% accuracy, a 99.90% F1 score, and a Kappa coefficient of 99.96%, indicating near-perfect classification.

(15) $$M_{Proposed}=f_{CNN}(X)+f_{Transformer}(X)+f_{Dual}(X)$$

This study confirms that the fusion of CNN and Transformer architectures, coupled with dual processing, leads to superior classification accuracy and reliability. Table 8 shows the performance comparison of individual models with the proposed model.

#### 5.5.1. Computational Time Analysis

Table 9 presents the performance metrics of different deep learning models on remote sensing image classification. It compares ResNetV2, Vision Transformer (ViT), and hybrid models (ResNetV2+ViT) in terms of training samples (m), validation samples (m), and computational cost (FLOPs). The RSI-CB256 dataset shows increasing FLOPs and training data requirements as models become more complex, with the dual-stream ResNetV2+ViT model requiring the most resources. The table compares four models ResNetV2, ViT, ResNetV2+ViT, and ResNetV2+ViT (dual stream) on the RSI-CB256 dataset using key performance and resource metrics. The dual-stream model takes the longest training time (65 min) and highest GPU memory usage (6.4 GB) but also yields the most computational complexity (44 FLOPs). In contrast, ResNetV2 is the most efficient, requiring only 45 min for training and 4.2 GB of GPU memory. ViT and the combined models show moderate to high resource consumption, reflecting the trade-off between model complexity and computational cost.

#### 5.5.2. Cross-Sensor-Based Performance Analysis

**Figure 12:**
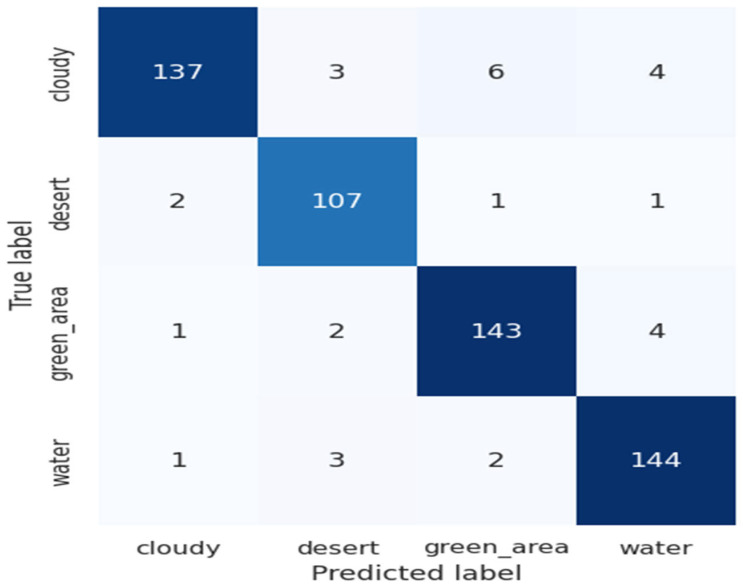
Confusion matrix for the cross-sensor dataset.

We performed cross-sensor-based performance analysis of the ResV2ViT. The ResV2ViT is trained on the Land Use Scene Classification Dataset (LUSCD), which has 21 classes and contains 10500 images. Furthermore, experimental conditions are kept the same as discussed in Section 4.1. For testing, we randomly selected 10% of data from the RSI-CB256 dataset, and the confusion matrix is presented in Figure 12. This confusion matrix shows the classification performance of a model on four classes: cloudy, desert, green_area, and water. The model performs well, with high true positive counts along the diagonal (e.g., 137 for cloudy, 144 for water). Misclassifications are minimal, such as six cloudy images predicted as green_area or three water images predicted as desert, indicating strong overall accuracy and effective class differentiation.

From the confusion matrix shown in Figure 12, we calculated performance measures shown in Table 10. The table presents the classification performance metrics for four classes: cloudy, desert, green_area, and water. Each class shows high precision, recall, and F1 score, indicating effective and balanced performance. For instance, desert has the highest precision (96.40%), while cloudy has the highest recall (97.16%). The F1 scores for all classes are above 94%, reflecting the model’s robustness. The overall accuracy is 94.65%, demonstrating the model’s strong capability in correctly classifying diverse land cover types.

**Figure 13:**
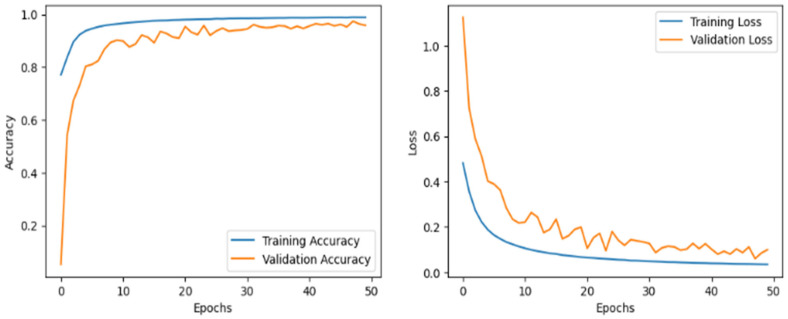
Accuracy and loss curve on the cross-sensor dataset.

We plotted the loss and accuracy curve shown in Figure 13. The graphs illustrate the model’s training and validation performance over 50 epochs. The left plot shows accuracy steadily increasing, with training accuracy nearing 100% and validation accuracy stabilizing around 95%, indicating effective learning. The right plot displays a consistent decrease in both training and validation loss, with slight fluctuations in validation loss, suggesting minimal overfitting. Overall, the model generalizes well and converges effectively, achieving high accuracy with low loss on both training and validation datasets.

**Figure 14:**
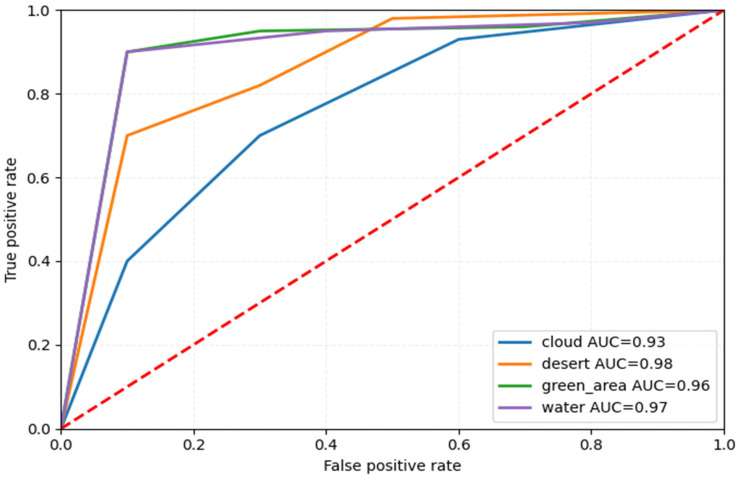
The AUC value of each class in the RSI-CB256 dataset.

Furthermore, we plotted the ROC plot shown in Figure 14. Figure 14 shows that the cloud class AUC value is the lowest due to the class having no availability in the training dataset LUSCD. At the same time, the desert class obtained a 0.98 AUC value. Furthermore, the green_area and water classes have AUC values of 0.96 and 0.97, respectively.

### 5.6. The Grad-CAM Based Performance Analysis

**Figure 15:**
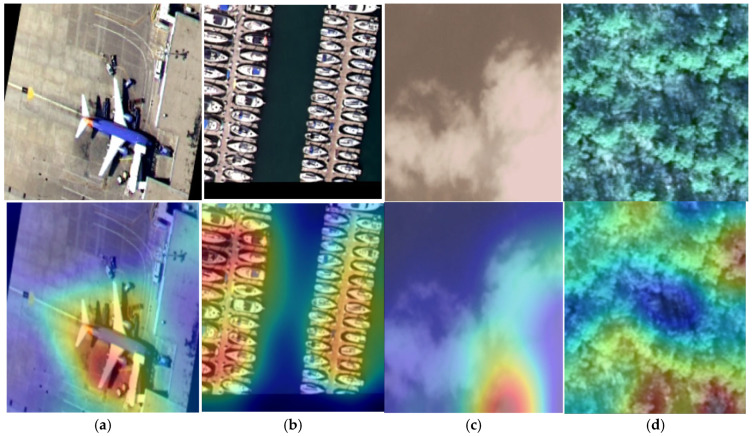
Sample image and corresponding Grad-CAM results: (**a**) airplane, (**b**) harbor, (**c**) cloud, and (**d**) forest.

We implemented Grad-CAM to visualize the decision process of the model on the datasets and some sample results are presented in Figure 15. In Figure 15, we can notice the proposed model focused on the region of interest in all classes. However, in the cloud class, some region is not fully utilized by the model. In addition, for the forest class, very few regions are not highlighted by the model.

## 6. Conclusions

This study introduces a hybrid deep learning model, ResV2ViT, which effectively integrates ResNet50 V2 and Vision Transformer (ViT) in a dual-stream architecture for remote sensing satellite image classification. By simultaneously leveraging two complementary streams, one for localized feature extraction using CNNs and another for global attention-based reasoning with Transformers, the model effectively captures both fine-grained spatial details and broader contextual relationships. The proposed dual-stream approach achieves state-of-the-art performance on two datasets, an RSI-CB256 dataset and a second dataset with 21 classes, with an average precision of 99.91%, an F1 score of 99.90%, and a precision of 99.90% for Dataset 1. The model also showed a 98.71% accuracy for the second dataset. These results highlight the model’s robustness and superiority over conventional deep-learning approaches. Despite its strong performance, the dual-stream hybrid architecture ResV2ViT model has certain limitations. The increased computational complexity due to the hybrid architecture may pose challenges for real-time applications and large-scale datasets.

Additionally, the dependency on pre-trained models and fine-tuning strategies requires further exploration to enhance generalizability across diverse remote sensing datasets. Future research can focus on optimizing the model’s efficiency through lightweight Transformer architectures and pruning techniques to reduce computational overhead. Moreover, investigating its applicability to multi-modal remote sensing data (e.g., hyperspectral and SAR imagery) could further enhance its real-world utility. Expanding the model for temporal change detection, integrating self-supervised learning, and deploying it in edge computing environments are promising directions for future advancements. By addressing these challenges, the ResV2ViT model can serve as a versatile and scalable solution for remote sensing applications, paving the way for improved geospatial analytics, environmental monitoring, and disaster management. Moreover, in future studies, we will utilize hyperspectral datasets such as Indian Pines, Pavia University, Salinas Valley, and Botswana for more accurate analysis. The ResV2ViT architecture will be slightly modified to handle hyperspectral spectral and spatial resolution. We will apply PCA to reduce the dimension of the hyperspectral data. After that, a lightweight 3D convolution block will be used to capture the spectral features. Finally, ResV2ViT will be utilized to explore the joint spectral and spatial information available for land cover classification.

## Figures and Tables

**Table 1:** Summary of the recent works.

References	Model	Hyperparameters	Dataset Used	Results
Sun et al. [5]	Fast motion object-detection algorithm	-	-	Accuracy = 93%, F1 score = 90%
Wang et al. [6]	RODNet (Radar Object-Detection Network)	Frame rate = 30FPS, Resolution = 1.6 MegaPixels	Camera Radar of the University of Washington (CRUW)	Precision = 86%, recall = 88%
Zhang et al. [7]	SATNet (Spectrum-Aware Transform Network)	Learning rate = 1 × 10^−5^, Batch size = 32, Training epoch = 50	Santa Barbara, Bay Area, Hermiston City	Accuracy = 0.9839, Kappa = 0.8883, precision = 0.8302, F1 score = 0.8969
Shi et al. [8]	DAHT-Net (Deformable Attention-Guided Hierarchical Transformer Network)	Computational cost = 12.65 min/epoch	LEVIR-CD, CDD, WHU-CD	Precision = 92.49%, recall = 93.18%, F1 score = 92.83%, accuracy = 98.93%
Gao et al. [9]	DSFT-Net (Deep Spatial Feature Transformation Network)	Learning rate = 0.001, Batch size = 8	UCAS-AOD, HRSC2016	mAP = 91.63%
Butler et al. [10]	Mask R-CNN	Epochs = 50, Learning rate = 0.002	COCO, Aerial-cars, Vehicle-Detection	mAP = 93.1%,
Xu et al. [11]	TCIANet (Transformer-Based Context Information Aggregation Network)	Momentum = 0.9, Weight decay = 0.0005, Initial learning rate = 0.01	CDD, LEVIR-CD, WHU	Precision = 95.98%, recall = 93.41%, F1 score = 95.68%, accuracy = 98.73%
Jiang et al. [12]	LFHNet (Lightweight Full-Scale Hybrid Network)	Momentum = 0.99, Weight decay = 5 × 10^−4^, Learning rate = 0.01, Training epochs = 200	LEVIR-CD, WHU-CD, DSIFN-CD	Precision = 92.75%, recall = 87.92%, F1 score = 90.27%, accuracy = 99.03%
Han et al. [13]	HANet (Hierarchical Attention Network)	Weight decay = 5 × 10^−4^, Learning rate = 5 × 10^−4^	WHU-CD, LEVIR-CD	Accuracy = 99.16%
Wan et al. [14]	CLDRNet (Category Context Learning-Based Difference Refinement Network)	Momentum = 0.99, Weight decay = 0.0005, Learning rates = 0.01, Batch size = 8	LEVIR-CD, BCDD, CDD	Recall = 96.16%, precision = 95.64%, F1 score = 95.90%, accuracy = 99.03%
Li et al. [15]	MDFENet	Batch size = 16, Learning rate = 0.005	LEVIR-CD, SYSU-CD	Accuracy = 91.06%
Sun et al. [16]	STCD Former	Epoch = 200	Farmland, Santa Barbara	Accuracy = 99.25%, Kappa = 95.99%
Jin et al. [17]	CR-DINO (Camera Radar-based DETR)	Epochs = 12, Batch size = 1, Learning Rate = 1 × 10^−4^, Number of queries = 900	nuScenes	mAP = 38.0, mAP = 41.7
Jia et al. [18]	Hybrid model of Transformer and CNN	Epochs = 200, 50 rounds of training	LEVIR-CD, BCDD t	MioU = 82.41%
Guo et al. [19]	TFIFNet (Transformer with Feature Interaction and Fusion Network)	Learning Rate = 0.001, Momentum = 0.9, Weight Decay = 0.0005	CLCD, SYSU-CD	Precision = 90.87%, recall = 87.08%, F1 score = 88.77%, accuracy = 92.29%
Tan et al. [20]	BD-MSA (Body Decouple Multiscale by Feature Aggregation)	BCE Loss as the loss function	DSIFN-CD, WHU-CD, S2Looking	Recall = 80.3%, precision = 88.01%, F1 score = 83.98%
Xiong et al. [21]	MLA-Net	Batch size = 16, Epochs = 50	LEVIR-CD, CLCD, WHU-CD	Accuracy = 99.08%, F1 score = 90.87%

**Table 2:** Notation Summary Table.

Symbol	Description
x_l_	Input to the lth residual layer
y_l_	Output of the residual unit after adding identity and transformation function
h(x_l_)	Identity mapping (h(x_l_) = x_l_)
F(x_l_, W_l_)	Residual function with learnable weights W_l_
T_i_	Token generated from the iᵗʰ patch of the feature map
W_e_	Linear projection weight matrix for tokenization
b_e_	Bias term added during tokenization
T_i_^1D^	Flattened 1D token with position embedding
P_e_	Position embedding vector
q_1_	Token subset sent to the convolutional stream
q_2_	Token subset sent to the Transformer stream
W_c_, b_c_	Weights and bias for convolutional block
σ	Activation function (e.g., ReLU)
Q, K, V	Query, Key, and Value matrices for self-attention
W_q, W_k, W_v	Weight matrices to compute Q, K, V from q_2_
d_k_	Dimension of key vectors (used for attention scaling)
Z	Combined feature representation after feature fusion
GAP(·)	Global Average Pooling
W_fc, b_fc	Weights and bias of the final classification layer
P	Predicted class probabilities
y_i_	True label for class i
y_p	Predicted probability for class i
z_i_	Logit (raw score) for class i before softmax
L	Loss function value (categorical cross-entropy)

**Table 3:** Performance comparison of 5 folds of the proposed model.

Fold	Precision (%)	Recall (%)	F1 Score (%)	Accuracy (%)
Fold 5	100	100	100	100
Fold 4	99.85	99.92	99.90	99.91
Fold 3	99.91	99.91	99.91	99.91
Fold 2	99.91	99.91	99.91	99.91
Fold 1	99.84	99.80	99.82	99.82
Average	99.90	99.90	99.90	99.91

**Table 4:** Performance parameters of ResV2Vit model on the second dataset.

Class	Precision (%)	Recall (%)	F1 Score (%)	Overall Accuracy (%)
Agricultural	100	96.97	98.46	98.71
Airplane	100	100	100	
Baseballdiamond	100	100	100	
Beach	94.74	100	97.3	
Buildings	97.35	99.1	98.21	
Chaparral	100	100	100	
Denseresidential	96.15	98.04	97.09	
Forest	97.89	100	98.94	
Freeway	99.17	100	99.58	
Golfcourse	98.13	100	99.06	
Harbor	100	100	100	
Intersection	98.95	96.91	97.92	
Mediumresidential	99.07	95.54	97.27	
Mobilehomepark	98.91	98.91	98.91	
Overpass	98.11	99.05	98.58	
Parkinglot	100	100	100	
River	98.13	99.06	98.59	
Runway	97.83	100	98.9	
Sparseresidential	98.04	98.04	98.04	
Storagetanks	100	95.83	97.87	
Tenniscourt	100	96.33	98.13	
Average	98.69	98.75	98.71	

**Table 5:** Performance comparison of various models on the RSI-CB256 dataset.

References	Model	Results
Jayasree et al. [22]	ResNet50	Accuracy = 96.53%
Li et al. [23]	ResNet50	Accuracy = 95.02%
Scott et al. [24]	ResNet50	Accuracy = 99.38%
Yogesh et al. [25]	VGG-16	Accuracy = 99%
Kaur et al. [26]	VGG-19	Accuracy = 93% F1 score = 93.25%
Tumpa et al. [27]	LPCNN-SVM	Accuracy = 99.8% Precision = 99.67%
Ulla et al. [28]	SATNet	Accuracy = 99.15% F1 score = 99%
Tehsin et al. [29]	ResNet	Accuracy = 97.7% Kappa = 96.9%
Sharma et al. [30]	EfficientNet + SVM	Accuracy = 97.11%
Liu et al. [31]	FCHNNN	Accuracy = 98%
Proposed	ResV2ViT	Accuracy = 99.91%

**Table 6:** Performance comparison of various models on the Land Use Scene Classification dataset.

References	Model	Accuracy (%)	Dataset Used
Qi et al. [32]	BoVW	98.00	Land Use Scene Classification Dataset
Zhao et al. [33]	CCM-BoVW	86.64	
Wu et al. [34]	Deep Filter Banks	90.40	
Li et al. [35]	Best Activation Model (BAM)	99.00	
Hu et al. [36]	DSR	97.47	
Yao et al. [37]	Large Random patch (LRP)	79.40	
Fan et al. [38]	Unsupervised Feature Learning	89.05	
Chen et al. [39]	PSR	95.00	
Hu et al. [40]	UFL-SC	90.26	
Proposed	ResV2ViT	98.71	

**Table 7:** Comparison of various models on different datasets.

Reference	Model	Dataset Used	Results (%)
Song et al. [41]	ResNeXt	NWPU-RESISC45	Accuracy = 94.12
Abba et al. [42]	InceptionV4	RSI-CB256	Accuracy = 96.98 Precision = 96.98 Recall = 96.98 F1 score = 96.98
Saetchnikov et al. [43]	YOLOV9	Airbus	Average Precision = 98.7%
Le et al. [44]	ViT	EuroSAT	Accuracy = 98.76 Precision = 98.77
Huang et al. [45]	SwinT	DFC2018	Accuracy = 80.15

**Table 8:** Performance comparison of individual models with the proposed model.

Model	Precision (%)	Recall (%)	F1 Score (%)	Accuracy (%)	Kappa (%)
ResNetV2	98.60	98.55	98.57	98.75	97.90
ViT	99.05	99.05	99.05	99.10	98.50
ResNetV2+ViT	99.40	99.40	99.40	99.45	99.10
Proposed	99.90	99.90	99.90	99.91	99.96

**Table 9:** Time analysis of different models on two datasets.

Dataset	Model	Training (m)	Validation (m)	Flops	GPU Memory Usage (GB)
	ResNetV2	45	4	25	4.2
	ViT	55	5	30	5.5
RSI-CB256	ResNetV2+ViT	60	5	40	6.1
	ResNetV2+ViT (dual stream)	65	5	44	6.4

**Table 10:** Performance measures for cross-sensor data.

Class	Precision (%)	Recall (%)	F1 Score (%)	Overall Accuracy (%)
Cloudy	91.33	97.16	94.16	
Desert	96.40	93.04	94.69	94.65
Green_area	95.33	94.08	94.70	
Water	96.01	94.12	95.05	

## Data Availability

Dataset used in the study can be downloaded from https://www.kaggle.com/datasets/apollo2506/landuse-scene-classification, last accessed: 15 January 2025. https://www.kaggle.com/datasets/mahmoudreda55/satellite-image-classification, last accessed: 18 January 2025.
